# Antimalarial properties and molecular docking analysis of compounds from *Dioscorea bulbifera* L. as new antimalarial agent candidates

**DOI:** 10.1186/s12906-021-03317-y

**Published:** 2021-05-18

**Authors:** Prapaporn Chaniad, Mathirut Mungthin, Apirak Payaka, Parnpen Viriyavejakul, Chuchard Punsawad

**Affiliations:** 1 School of Medicine, Walailak University, Nakhon Si Thammarat, 80160 Thailand; 2 Department of Parasitology, Phramongkutklao College of Medicine, Bangkok, 10400 Thailand; 3 School of Science, Walailak University, Nakhon Si Thammarat, 80160 Thailand; 4 Department of Tropical Pathology, Faculty of Tropical Medicine, Mahidol University, Bangkok, 10400 Thailand

**Keywords:** Malaria, Anti-malarial activity, *Dioscorea bulbifera* L*.*, *Plasmodium falciparum*, Molecular docking

## Abstract

**Background:**

At present, the emergence and spread of antimalarial drug resistance has become a significant problem worldwide. There has been a challenge in searching for natural products for the development of novel antimalarial drugs. Therefore, this study aims to evaluate compounds from *Dioscorea bulbifera* responsible for antimalarial properties and investigate potential interactions of the compounds with *Plasmodium falciparum* lactate dehydrogenase (*Pf*LDH), an essential glycolytic enzyme in the parasite’s life cycle.

**Methods:**

An in vitro study of antimalarial activity against chloroquine (CQ)-resistant *Plasmodium falciparum* (K1 strain) and CQ-sensitive *P. falciparum* (3D7 strain) was performed using the ^3^H-hypoxanthine uptake inhibition method. The cytotoxic effects of the pure compounds were tested against Vero cells using a 3-(4,5-dimethylthiazol-2-yl)-2,5-diphenyltetrazolium bromide (MTT) assay. The interactions of the compounds with the *Pf*LDH active site were additionally investigated using a molecular docking method.

**Results:**

Quercetin (6) exhibited the highest antimalarial activity against the *P. falciparum* K1 and 3D7 strains, with IC_50_ values of 28.47 and 50.99 μM, respectively. 2,4,3′,5′-Tetrahydroxybibenzyl (9), 3,5-dimethoxyquercetin (4) and quercetin-3-*O*-β-D-galactopyranoside (14) also possessed antimalarial effects against these two strains of *P. falciparum*. Most pure compounds were nontoxic against Vero cells at a concentration of 80 μg/ml, except for compound 9, which had a cytotoxic effect with a CC_50_ value of 16.71 μM. The molecular docking results indicated that 9 exhibited the best binding affinity to the *Pf*LDH enzyme in terms of low binding energy (− 8.91 kcal/mol) and formed strong hydrogen bond interactions with GLY29, GLY32, THR97, GLY99, PHE100, THR101 and ASN140, amino acids as active sites. In addition, 6 also possessed remarkable binding affinity (− 8.53 kcal/mol) to *Pf*LDH by interacting with GLY29, ILE31, ASP53, ILE54, THR97 and THR101.

**Conclusion:**

Quercetin is a major active compound responsible for the antimalarial activity of *D. bulbifera* and is an inhibitor of *Pf*LDH. These findings provide more evidence to support the traditional use of *D. bulbifera* for malaria treatment. Structural models of its interactions at the *Pf*LDH active site are plausibly useful for the future design of antimalarial agents.

## Background

Malaria remains one of the life-threatening infectious diseases in tropical and subtropical regions of the world [1]. Of the five species of *Plasmodium* parasites that cause human malaria, *Plasmodium falciparum* is the most pathogenic species with the greatest likelihood of drug resistance [2, 3]. According to the World Malaria Report, there were an estimated 228 million cases of malaria and 405,000 deaths worldwide in 2018 [4]. At present, artemisinin-based combination therapies (ACTs) are the first-line treatment that has been recommended by the World Health Organization (WHO) for uncomplicated falciparum malaria in all endemic countries. Unfortunately, the emergence and spreading of artemisinin (ART)-resistant *P. falciparum* has already been reported in Southeast Asian countries, including Thailand, Africa and many other malaria endemic countries [5, 6]. The lack of an effective vaccine for malaria prevention and the widespread use of multidrug-resistant *P. falciparum* [7] have led to the urgent need to identify lead compounds and develop new alternative antimalarial drugs to possibly avoid problems related to drug resistance [8].

*Plasmodium falciparum* lactate dehydrogenase (*Pf*LDH) is an essential enzyme in the parasite’s life cycle for survival and growth. It controls the production of adenosine triphosphate (ATP) by catalyzing the conversion of lactate to pyruvate in the final step of the glycolytic pathway during the anaerobic erythrocytic stages of the *P. falciparum* life cycle [9]. The inhibition of *Pf*LDH leads to parasite death, suggesting a potential antimalarial target [10]; therefore, this enzyme is an attractive target for the design and discovery of antimalarial drugs.

Medicinal plants are a potential source for the discovery and development of new drugs since they contain various metabolites with a great variety of structures and pharmacological activities [11]. The widespread use of medicinal plants in the treatment of malaria with the discovery of two antimalarial drugs, quinine from the bark of a cinchona tree and artemisinin from *Artemisia annua* L., which are used worldwide [12, 13]. Therefore, in the search for drug candidates, medicinal plants are an alternative potential source to provide new antimalarial agents.

*Dioscorea bulbifera* L. is a traditional medicinal plant that is used for the treatment of malaria, diarrhea, diabetes, sore throat, gastric cancer, and wound infections and is also used in longevity preparations [14, 15]. It belongs to the Dioscoreaceae family, which is commonly known as air potato. Various extracts of this plant have been reported to possess various pharmacological effects, such as analgesic, anti-inflammatory [16], antioxidant [17], antimicrobial [18], antidiabetic [15], antihyperglycemic, antidyslipidemic [19] and anti-HIV-1 integrase activities [20, 21]. Remarkably, there have been no reports of any antimalarial activity from *D. bulbifera* until now. Therefore, this study aims to identify compounds from *D. bulbifera* responsible for antimalarial properties and investigate potential interactions of the compounds with *Pf*LDH, a target enzyme associated with the life cycle of malaria.

## Materials and methods

### Parasite culture and maintenance

Chloroquine (CQ)-resistant *P. falciparum* (K1 strain) and CQ-sensitive *P. falciparum* (3D7 strain) were kindly provided by the Department of Parasitology, Phramongkutklao College of Medicine, Thailand. The culture of *P. falciparum* malaria parasites was continuously performed using standard methods [22] with some modifications. RPMI-1640 medium was supplemented with noninfected type O-positive red blood cells (2% hematocrit), 2 mg/ml sodium bicarbonate, 10 μg/ml hypoxanthine (Sigma-Aldrich, New Delhi, India), 4.8 mg/ml HEPES (Himedia, Mumbai, India), 10% human AB serum and 2.5 μg/ml gentamicin (Sigma-Aldrich, New Delhi, India) [23].

### Extraction and isolation of compounds from plant material

Bulbils of *D. bulbifera* were collected from Uttaradit Province, Thailand, in 2011. The botanical material was identified by a botanist of the Forest Herbarium, Wildlife and Plant Conservation, Thailand. The plant specimen has been deposited in the Department of Pharmacognosy and Pharmaceutical Botany, Faculty of Pharmaceutical Sciences, Prince of Songkla University, Hat-Yai, Songkhla, Thailand with a voucher specimen of SKP 062040201.

**Fig. 1:**
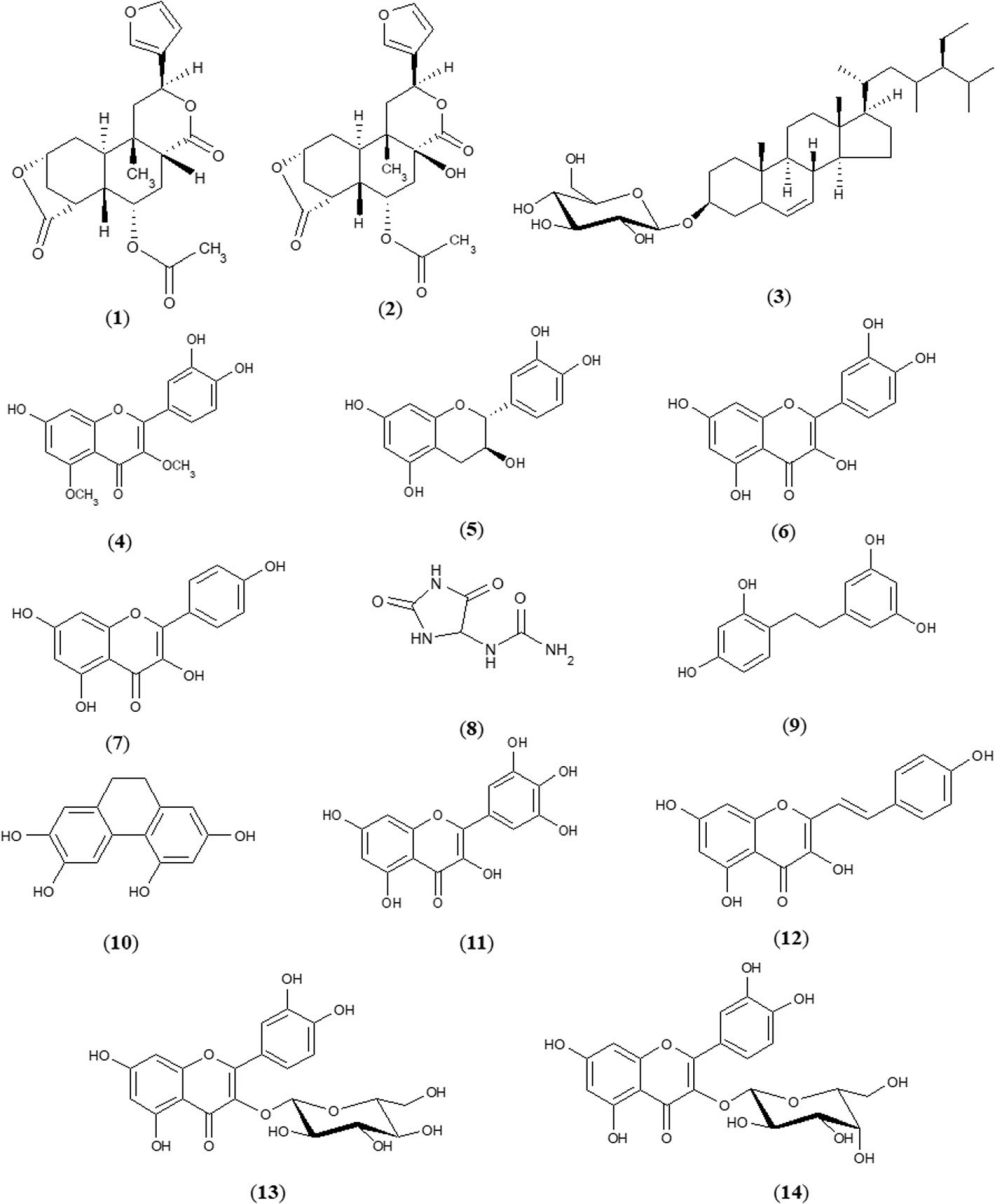
The structures of compounds isolated from *D. bulbifera* bulbils

According to previous reports by our research group, compounds 1–14 were purified from the ethanol extract of *D. bulbifera* bulbils by chromatography techniques and elucidated by spectroscopic methods [20, 21]. Briefly, ethanol extract by maceration method was successively partitioned with various solvents to give chloroform, ethyl acetate and water fractions. The chloroform fraction was separated by vacuum liquid chromatography (VLC), column chromatography (CC), preparative thin layer chromatography (PTLC) and Sephadex LH-20 to give seven compounds, including 8-epidiosbulbin E acetate (1), 15,16-epoxy-6α-*O*-acetyl-8β-hydroxy-19-nor-clero-13(16),14-diene-17,12;18,2-diolide (2), sitosterol-β-D-glucoside (3), 3,5-dimethoxy quercetin (4), (+)-catechin (5), quercetin (6) and kaempferol (7). The ethyl acetate fraction was separated by VLC, CC and Sephadex LH-20 to provide allantoin (8), 2,4,3′,5′-tetrahydroxybibenzyl (9), 2,4,6,7-tetrahydroxy-9,10-dihydrophenanthrene (10), myricetin (11) and 5,7,4′-trihydroxy-2-styrylchromone (12). In addition, the water fraction was fractionated on a Diaion HP-20 column and further isolated through an RP-18 column and Sephadex LH-20 to give quercetin-3-*O*-β-D-glucopyranoside (13) and quercetin-3-*O*-β-D-galactopyranoside (14). The chemical structures of the compounds were characterized by EI-MS and NMR spectroscopic methods, including ^1^H-NMR, ^13^C-NMR, DEPT, COSY, HMQC, HMBC and comparison with data in the literature. In the present study, the extracts and 14 compounds (1–14) from a previous study were investigated for antimalarial activity (Fig. 1).

### Antimalarial activity assay

Antimalarial activity of extracts and compounds from *D. bulbifera* against *P. falciparum* K1 and 3D7 strains were assessed by measuring ^3^H-hypoxanthine incorporated in parasite nucleic acids using the modified technique of Desjadins et al. [24]. The red cell suspension at 1–2% hematocrit containing 1% ring stage *P. falciparum-*infected red blood cells was incubated with various concentrations of extracts (2.50–80 μg/ml) and pure compounds (2.5–80 μM) in 96-well culture plates. Parasites were incubated at 37 °C for 24 h, then ^3^H-hypoxanthine (25 μl of 0.025 μCi/μl) was added to each well, and the plates were maintained under conditions of 93% N_2_, 3% O_2_ and 4% CO_2_ at 37 °C for an additional 24 h. The samples were transferred to a glass fiber filter (Wallac, Turku, Finland). ^3^H-Hypoxanthine uptake was then assessed using a MicroBeta TriLux Liquid Scintillation Counter (PerkinElmer, USA). Each assay condition was performed in triplicate independently. The concentration of samples that inhibited the uptake of ^3^H-hypoxanthine by 50% (IC_50_) assessed by log dose response curve using WinNonlin software version 4.1 (Pharsight Corporation, CA) was used as a marker of antimalarial effect potency. Artesunate at concentrations ranging from 0.1–20 nM and chloroquine at concentrations ranging from 2.0–250 nM were used as positive controls.

### In vitro assessment of cytotoxicity

The toxicity of *D. bulbifera* extracts and isolated compounds was assessed by 3-(4,5-dimethythiazol-2-yl)-2,5-diphenyl tetrazolium bromide (MTT) assay according to a previous method [23]. Briefly, Vero cells (Elabscience, Wuhan, Hubei, China) were seeded into 96-well plates at a density of 10^4^ cell/ml and incubated for 24 h at 37 °C with 5% CO_2_. Cells were then treated with various concentrations of extracts and compounds for 48 h at 37 °C with 5% CO_2_. Doxorubicin (Sigma-Aldrich, New Delhi, India) was used as a toxic control. Subsequently, MTT solution was added to each well, and the plate was incubated for 2 h in a CO_2_ incubator. The medium was then removed, and 100 μl of DMSO was added to each well. Finally, the optical density was determined at a wavelength of 590 nm using a microplate reader. The assay was performed in duplicate.

### Molecular docking

#### *Pf*LDH structure preparation

The 3D structure of *Pf*LDH (residues ALA18-ALA329) in complex with β-nicotinamide adenine dinucleotide phosphate disodium salt (NADH) and oxamate was obtained from the Protein Data Bank (PDB code 1LDG). The *Pf*LDH structure was prepared using AutoDock Tools. The missing residues were incorporated. All water molecules and oxamate, a competitive inhibitor of the binding of pyruvate to LDH and NADH cofactor, were removed so that a new ligand could enter the active site [10].

#### Ligand molecule preparation

The 3D structures of compounds 1–14, artesunate and chloroquine were generated using the HyperChem Professional 8.0 program. (Hypercube Inc., Gainesville, FL). Each structure was geometrically optimized using the semiempirical PM3 method. Subsequently, Gasteiger charges were assigned to the ligands using AutoDock Tools to model the appropriate structures for docking calculations.

### Molecular docking analysis

Molecular docking calculations were performed according to a previous method [25] using the AutoDock 4.2 program (Hypercube Inc., Gainesville, FL). The *Pf*LDH active site was selected as the ligand binding site. A grid box size of 60 × 60 × 60 Å^3^ was generated and centered on 32, 30 and 32 A° for x, y, and z, respectively, with a grid spacing of 0.375 Å. The Lamarckian genetic algorithm (LGA) was performed with the rigid receptor molecule to search for the best conformers. The docking run was set at a maximum of 100 conformations for each ligand. The population size was set at 150, the maximum number of energy evaluations was increased to 2,500,000, and the genetic generation was 100,000. The other parameters were set to default values of AutoDock 4.2. The docking methodology was evaluated by redocking to achieve more accurate results. The lowest binding energy conformation of the most populated cluster was considered the best-docked conformation that was chosen for analysis of the hydrogen bond interactions [20]. The 3D H-bond interactions between compounds and the binding site of the enzyme were generated by the UCSF Chimera 1.14 program, and hydrophobic interactions were evaluated using the protein ligand interaction profiler (PLIP) [26].

## Results

### Antimalarial property against *P. falciparum* K1 strain

The ethanol extract of *D. bulbifera* exhibited good activity against the *P. falciparum* K1 strain with an IC_50_ value of 15.8 μg/ml, while the water extract showed weak activity (IC_50_ >  80 μg/ml). Among 14 tested compounds, quercetin (6) possessed the highest effect with good activity (IC_50_ = 28.47 μM) and showed 97.01% inhibition at a concentration of 80 μM, followed by 2,4,3′,5′-tetrahydroxybibenzyl (9, IC_50_ = 39.99 μM). 3,5-Dimethoxyquercetin (4), quercetin-3-*O*-β-D-galactopyranoside (14), 2,4,6,7-tetrahydroxy-9,10-dihydrophenanthrene (10) and kaempferol (7) also exhibited antimalarial effects with moderate activity with IC_50_ values of 44.03, 48.33, 58.34 and 62.45 μM, respectively (Table 1), whereas the other compounds were apparently weakly active (IC_50_ >  80 μM).

**Table 1:** Antimalarial properties against *P. falciparum* K1 and 3D7 strains and the cytotoxicity of extracts and isolated compounds from *D. bulbifera*

Samples	IC_**50**_ (μM/nM^d^)		CC_**50**_ (μM)
	K1	3D7	
Ethanol extract^c^	15.8 ± 3.18	> 80	> 80
Water extract^c^	> 80	> 80	> 80
8-Epidiosbulbin E acetate (1)	> 80	> 80	> 80
15,16-Epoxy-6α-*O*-acetyl-8β-hydroxy-19-nor-clero-13 (16),14-diene-17,12;18,2-diolide (2)	> 80	> 80	> 80
Sitosterol-β-D-glucoside (3)	> 80	> 80	> 80
3,5-Dimethoxyquercetin (4)	44.03 ± 1.47^a,b^	70.79 ± 2.32^a,b^	> 80
(+)-Catechin (5)	> 80	> 80	> 80
Quercetin (6)	28.47 ± 0.90^a,b^	50.99 ± 7.28^a,b^	> 80
Kaempferol (7)	62.45 ± 1.33^a,b^	> 80	> 80
Allantoin (8)	> 80	> 80	> 80
2,4,3′,5′-Tetrahydroxybibenzyl (9)	39.99 ± 2.50^a,b^	58.85 ± 4.31^a,b^	16.71
2,4,6,7-Tetrahydroxy-9,10-dihydrophenanthrene (10)	58.34 ± 1.96^a,b^	> 80	> 80
Myricetin (11)	> 80	> 80	> 80
5,7,4′-Trihydroxy-2-styrylchromone (12)	> 80	> 80	> 80
Quercetin-3-*O*-β-D-glucopyranoside (13)	> 80	> 80	> 80
Quercetin-3-*O*-β-D-galactopyranoside (14)	48.33 ± 1.21^a,b^	68.93 ± 4.31^a,b^	> 80
Chloroquine^d^	103.2 ± 4.50	9.91 ± 0.56	ND
Artesunate^d^	0.53 ± 0.04	1.81 ± 0.19	ND
Doxorubicin	ND	ND	1.96 ± 0.11

*ND* not determined

^a^Statistically significant difference between chloroquine and the sample, *p* < 0.05 (mean ± S.D. of three determinations)

^b^Statistically significant difference between artesunate and the sample, *p* < 0.05 (mean ± S.D. of three determinations)

^c^Concentration of treated samples and IC_50_ unit expressed in μg/ml

^d^Concentration of positive control and IC_50_ unit expressed in nM

### Antimalarial property against *P. falciparum* 3D7 strain

The results for the *P. falciparum* 3D7 strain were similar in the K1 strain; quercetin (6) also possessed the highest antimalarial activity (IC_50_ = 50.99 μM), followed by 2,4,3′,5′-tetrahydroxybibenzyl (9), quercetin-3-*O*-β-D-galactopyranoside (14) and 3,5-dimethoxyquercetin (4), with IC_50_ values of 58.85, 68.93 and 70.79 μM, respectively (Table 1).

### In vitro cytotoxicity

Most pure compounds revealed nontoxic effects on Vero cells at a concentration of 80 μg/ml except for 2,4,3′,5′-tetrahydroxybibenzyl (9), which had cytotoxic effects with a 50% cytotoxic concentration (CC_50_) value of 16.71 μM (Table 1).

### Molecular docking

**Fig. 2:**
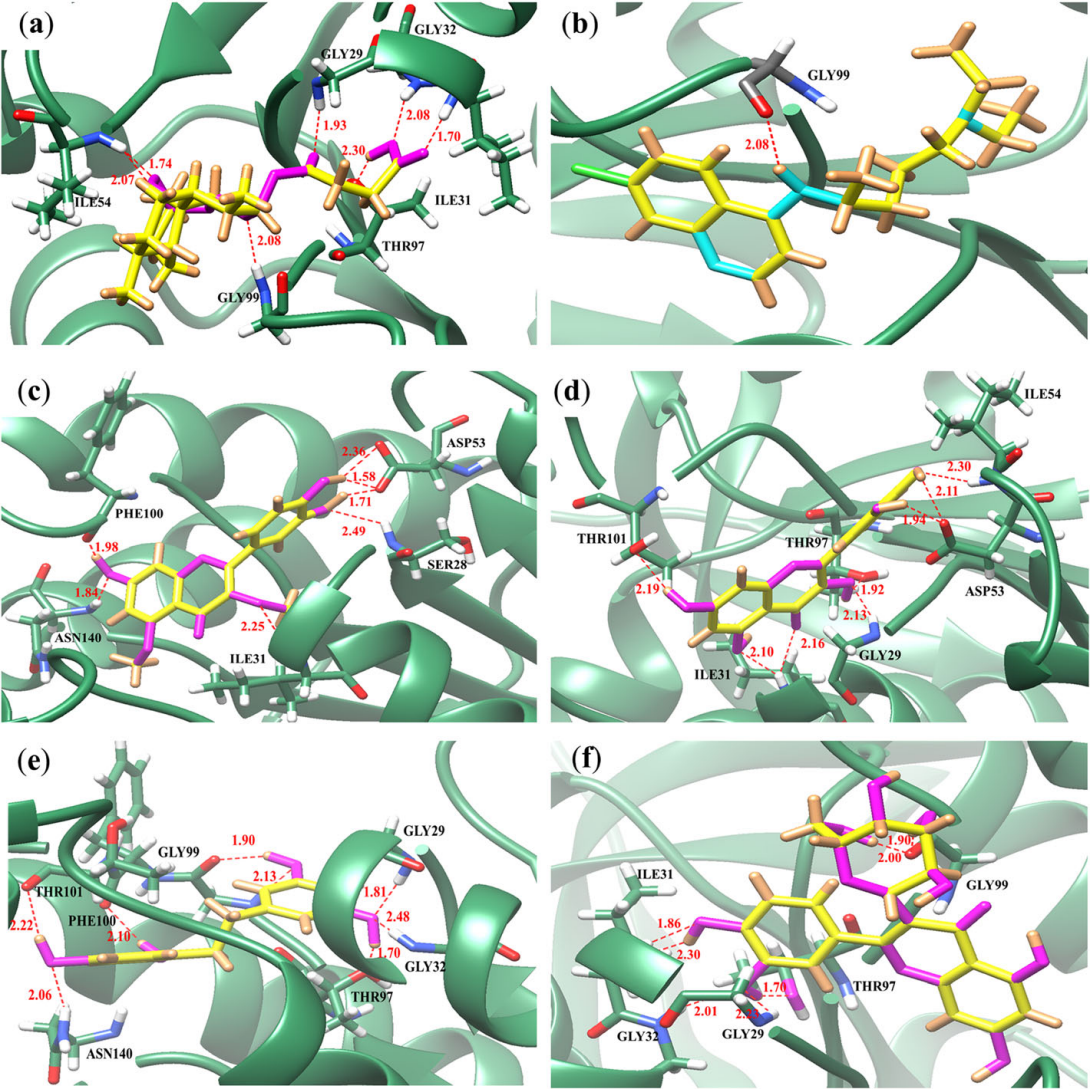
Predicted binding modes of active compounds, artesunate and chloroquine with *Pf*LDH. The backbones of *Pf*LDH enzyme are depicted in green ribbon models and all hydrogen bonding residues are shown as stick models and labeled by heteroatom; white for H, blue for N, red for O. Compounds are labeled by heteroatom; yellow for C, brown for H, cyan for N, magenta for O. Hydrogen bond interactions are shown as red dash lines and represent bond length in angstrom (A°). **a** Artesunate. **b** Chloroquine. **c** 3,5-Dimethoxyquercetin. **d** Quercetin. **e** 2,4,3′,5′-Tetrahydroxybibenzyl. **f** Quercetin-3-*O*-β-D-galactopyranoside

To predict the potential interactions of compounds with *Pf*LDH enzyme targets, molecular docking calculations were performed. The binding energy and amino acid residues of *Pf*LDH that interacted with each compound and the hydrogen bonds are given in Table 2. The binding energy with a higher negative value corresponds to a more stable interaction between the compound and target enzyme. To predict the binding modes of active compounds with *Pf*LDH and identify the interacting amino acid residues, the 2D interactions of the top four active compounds (4, 6, 9 and 14) with *Pf*LDH were created, as shown in Fig. 2. Among the 14 compounds, 2,4,3′,5′-tetrahydroxybibenzyl (9), which possessed a good antimalarial effect (IC_50_ = 39.99 μM), exhibited the best binding affinity to *Pf*LDH in terms of a low binding energy of − 8.91 kcal/mol; however, its binding energy was lower than that of artesunate (− 11.21 kcal/mol). It is predicted to strongly interact with eight hydrogen bonds with GLY29, GLY32, THR97, GLY99, PHE100, THR101 and ASN140 (Fig. 2e). Additionally, compound 9 was stabilized through hydrophobic interactions with residues THR101, LEU112 and ASN140 (Table 2). For artesunate, the potent antimalarial drug interacted with ILE31, GLY29, GLY32, ILE54, THR97, and GLY99 of the *Pf*LDH active site (Fig. 2a) and formed hydrophobic interactions with VAL26, ILE31, PHE52, ILE54, ALA98, THR101, and ILE119 (Table 2). While chloroquine possessed a weak interaction, it formed only one hydrogen bond with GLY99 with a binding energy of − 6.65 kcal/mol. (Fig. 2b) as well as hydrophobic interactions with VAL26, ILE31, PHE52, THR101 and ILE119.

**Table 2:** The binding energy and interacting amino acid residues of compounds from *D. bulbifera* with *Pf*LDH

Compounds	Binding energy (kcal/mol)	H-bond interaction		Hydrophobic interaction	
		Number of interaction	Amino acid residues	Number of interaction	Amino acid residues
1	−7.10	4	ILE31^a^, ASN140, SER245	5	ILE31, THR97, THR101, VAL138, PRO250
2	−6.98	7	MET30^a^, ILE31, THR97, GLY99, ASN140^a^	0	–
3	−7.33	5	PHE100^a^, ASN140^a^, SER245	6	ILE31, ILE54^a^, VAL55, ALA98, ILE119
4	−7.55	7	SER28, ILE31, ASP53^b^, PHE100, ASN140	1	THR101
5	−7.66	8	ILE31, GLY32, GLY99, PHE100, THR101, ASN140^a^, SER245	2	THR97, THR101
6	−8.53	8	GLY29, ILE31^a^, ASP53^a^, ILE54, THR97, THR101	2	MET30, ALA98
7	−7.16	8	PHE100, ARG109, ASN140^a^, ASN166, ARG171, SER245, PRO246	6	THR101, TRP107^a^, ASN140, LEU167, ALA236
8	−5.09	9	GLY29, ILE31^a^, GLY32, THR97^a^, GLY99^b^	0	–
9	−8.91	8	GLY29, GLY32, THR97, GLY99^a^, PHE100, THR101, ASN140	3	THR101, LEU112, ASN140
10	−6.64	5	GLY29, ILE31, GLY32, THR97^a^	2	ILE31, THR101
11	−6.55	7	GLY29, GLY32, THR97, GLY99, PHE100, VAL138, PRO246	4	ILE31, THR97, THR101, VAL138
12	−7.89	6	GLY99^a^, PHE100, ASN140, SER245^a^	4	ILE31^a^, LEU112, ASN140
13	−6.88	6	PHE52, ASP53, ILE54, GLY99^a^, LYS118	9	VAL26, PHE52, ILE54^a^, ALA98, LYS118, ILE119, GLU122, ILE123
14	−7.86	7	GLY29, ILE31^a^, GLY32, THR97, GLY99^a^	3	ILE54, ALA98, THR101
Artesunate	−11.21	7	ILE31, GLY29, GLY32, ILE54^a^, THR97, GLY99	9	VAL26, ILE31, PHE52, ILE54^a^, ALA98, THR101, ILE119^a^
Chloroquine	−6.65	1	GLY99	6	VAL26, ILE31^a^, PHE52, THR101, ILE119

^a^Two interactions with amino acid residues

^b^Three interactions with amino acid residues

Interestingly, quercetin (6), which possessed the strongest antimalarial activity against *P. falciparum* K1 and 3D7 strains in vitro*,* also showed a remarkable binding affinity to *Pf*LDH with a binding energy of − 8.53 kcal/mol. Its structure fits well to the active site of *Pf*LDH, as demonstrated in Fig. 2d. All five hydroxyl groups of quercetin formed eight hydrogen bonds with the enzyme target, including GLY29, ILE54, THR97, and THR101, and strongly interacted with ILE31 and ASP53 with two hydrogen bonds each. Furthermore, quercetin (6) also formed hydrophobic interactions with MET30 and ALA98. 3,5-Dimethoxyquercetin (4), which possessed moderate activity against the *P. falciparum* K1 strain (IC_50_ = 44.03 μM), had a slightly lower binding energy (− 7.55 kcal/mol) and formed seven hydrogen bonds with ILE31, ASP53, PHE100, ASN140 and SER28 (Fig. 2c). A remarkable result was also observed with kaempferol (7), a compound that showed moderate antimalarial activity against the *P. falciparum* K1 strain (IC_50_ = 62.45 μM). This compound strongly interacted with residues in the active site region of *Pf*LDH with a binding energy of − 7.16 kcal/mol. It formed eight hydrogen bonds with PHE100, ARG109, ASN140, ASN166, ARG171, SER245 and PRO246. Kaempferol (7) formed additional hydrophobic interactions with THR101, TRP107, ASN140, LEU167 and ALA236.

**Fig. 3:**
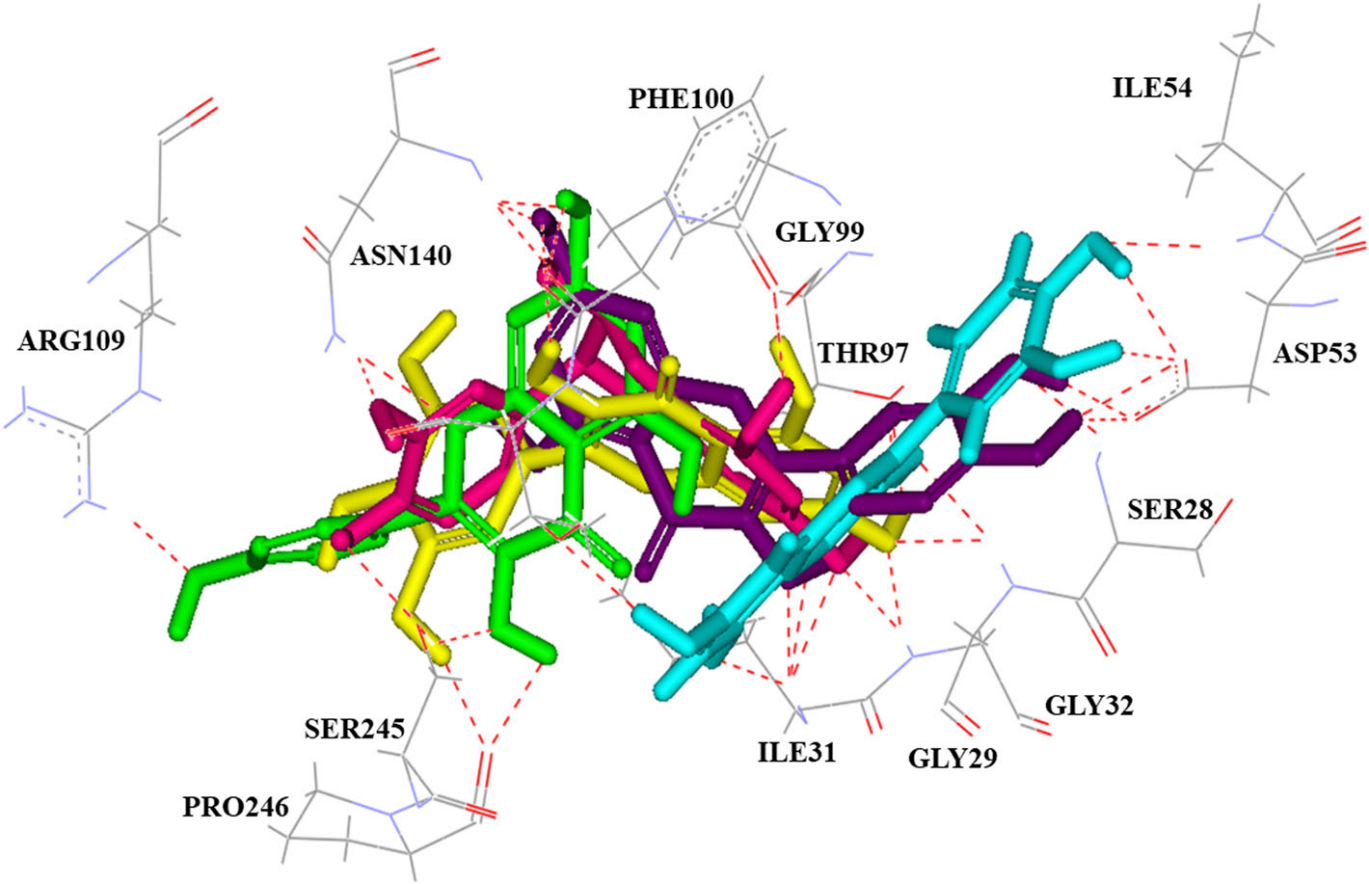
Superimposed structures of the docked conformations of flavonoid compounds with amino acid residues of *Pf*LDH. 3,5-Dimethoxyquercetin (4; purple), (+)-catechin (5; magenta), quercetin (6; cyan), kaempferol (7; green) and myricetin (11; yellow). Hydrogen bond interactions are shown as red dashed lines

In particular, compounds 4, 5, 6, 7 and 11, which were identified as flavonoids and possessed different antimalarial effects with IC_50_ values of 44.03, > 80, 28.47, 62.45 and >  80 μM, respectively, also presented different binding modes, while their binding locations were similar (Fig. 3). Quercetin (6), the strongest compound, showed a different binding pattern than the other compounds. It directly pointed to ASP53 and interacted tightly with two hydrogen bonds. Quercetin-3-*O*-β-D-glucopyranoside (13) and quercetin-3-*O*-β-D-galactopyranoside (14) were identified as quercetin glycosides; they showed different binding energies of − 6.88 and − 7.86 kcal/mol, respectively, and interacted with different amino acid residues. Compound 14, which exhibited higher activity against the K1 strain (IC_50_ = 48.88 μM) than compound 13 (IC_50_ >  80 μM), interacted with residues GLY29, ILE31, GLY32, THR97, GLY99 through seven hydrogen bonds (Fig. 2f), while 13 formed six hydrogen bonds with PHE52, ASP53, ILE54, GLY99 and LYS118. These docking results were related to its antimalarial activity, which was determined by an in vitro hypoxanthine assay model. Based on the results of the antimalarial effect against the *P. falciparum* K1 strain in the in vitro model, compounds 1, 2, 3, 5, 8, 11, 12 and 13, which are considered inactive compounds (IC_50_ >  80 μM), were mostly weaker interactions with the *Pf*LDH enzyme than those of active compounds in terms of binding energy and number of hydrogen bonds.

## Discussion

The main obstacle of malaria treatment and control is the emergence of drug-resistant parasite strains, which include artemisinin, the first-line drug for treating uncomplicated multidrug-resistant falciparum malaria [27]. To resolve the problem of multidrug resistance, it is necessary to identify new alternative antimalarial agents with higher activity. In the present study, ethanol and water extracts along with 14 isolated compounds of *D. bulbifera* bulbils were investigated in *P. falciparum* K1 and 3D7 strains. The results revealed that the ethanol extract possessed a good antimalarial effect (IC_50_ = 15.8 μg/ml) against the K1 strain. Among the 14 tested compounds, quercetin, a flavonoid compound, was the most active on the two strains of *P. falciparum,* with IC_50_ values of 28.47 and 50.99 μM for the K1 and 3D7 strains, respectively. Notably, the antimalarial effect of all compounds against the K1 strain was higher than that of the 3D7 strain. This effect could be due to the difference in genetics between these two parasite strains [28].

Considering the effect of seven flavonoid compounds (4, 5, 6, 7, 11, 13, 14), the results showed that quercetin (6) possessed the most potent antimalarial effect against both K1 and 3D7 strains. It showed potent activity approximately 1.5- and 2-fold higher than that of 3,5-dimethoxyquercetin (4) and quercetin-3-*O*-β-D-galactopyranoside (14), respectively, which was observed in the K1 strain. Regarding the structure-activity relationships, quercetin (6), an aglycone bearing a catechol moiety in the B-ring and lacking a glycoside chain, exhibited relatively higher activity than its derivatives, as observed from the comparison of quercetin (6; IC_50_ = 28.47 μM) and 3,5-dimethoxyquercetin (4; IC_50_ = 44.03 μM) for the K1 strain. The result was also clearly observed when quercetin (6) was compared with quercetin glycosides (13 and 14, IC_50_ = 100 and 48.33 μM, respectively). These results are in agreement with a previous study reporting that quercetin exhibited antimalarial activity higher than that of quercetin glycosides to inhibit the growth of *P. falciparum* K1 and 3D7 strains [29]. Quercetin was found in a variety of fruits and vegetables and in medicinal plants, including *Ginkgo biloba*, *Hypericum perforatum*, *Sambucus canadensis*, *Aesculus indica*, and *Dendrobium officinale* [30, 31]. It has been reported to possess various biological activities, including anti-inflammatory [32], antioxidant [33], anticancer [34] and anti-HIV integrase activities [21]. Regarding the other *Dioscorea* species, *D. loureiri* and *D. membranacea* have been reported to exhibit antimalarial activity against both *P. falciparum* 3D7 and K1 strains [35].

In an attempt to investigate the mechanism of active compounds against *P. falciparum*, the binding mode of compounds with the target protein of *P. falciparum* was predicted using computational docking. Molecular docking is one of the most frequently used methods to predict the interaction of two molecules in structure-based drug design with a substantial degree of accuracy [36, 37]. In the present study, the *Pf*LDH enzyme was selected for study since it has been considered a potential molecular target for antimalarial drugs because it controls energy production in plasmodium. In addition, this enzyme is found in all five species that cause human malaria, including *P. falciparum*, *P. vivax*, *P. ovale*, *P. malariae* and *P. knowlesi* [38, 39]. The biological functions of *Pf*LDH and human LDH are very similar, but their amino acid sequences are less similar. Therefore, the selective targeting of this glycolytic enzyme in *Pf*LDH may not disturb human LDH [9].

Regarding *P. falciparum* molecular targets, other *P. falciparum* targets have been proven to be major molecular drug targets of antimalarial drugs. *P. falciparum* dihydrofolate reductase (*Pf*DHFR)*,* a key enzyme in de novo folate biosynthesis, is considered a known target for malaria. As a result, antifolate antimalarial drugs (pyrimethamine and cycloguanil) inhibit dihydrofolate reductase (DHFR) and interfere with folate metabolism, a pathway essential to malaria parasite survival [40]. Additionally, *Pf*ATP6, the SERCA-type Ca^2+^-ATPase enzyme present in the malarial parasite, has been identified as the molecular target for artemisinin, curcumin and curcumin derivatives [41].

Regarding docking, the predicted binding energy is calculated. A more negative binding energy indicates stronger binding [10]. Docking results in accordance with the in vitro results showed that quercetin, the most potent antimalarial activity against *P. falciparum*, which is characterized by the presence of five hydroxyl groups at positions 3, 5, 7, 3′ and 4′, also possessed strong interactions with *Pf*LDH. It was found to have similar interactions to the standard artesunate, the structure fitted well to the active site, and all of its hydroxyl groups strongly interacted with residues GLY29, ILE31, ASP53, ILE54, THR97 and THR101. The oxygen of the hydroxyl group of this compound extensively contacts ILE31 and ASP53 by forming two hydrogen bonds each. Quercetin showed different binding patterns compared to other flavonoids. It directly pointed to ASP53 and tightly formed hydrogen bonds. These binding pattern differences as well as the high number of hydrogen bonds that formed with *Pf*LDH residues were possible reasons why this compound was active.

2,4,3′,5′-Tetrahydroxybibenzyl (9), an active compound from an in vitro study, was found to bind preferentially in a similar way to quercetin. All four hydroxyl groups on its structure formed eight hydrogen bonds with residues GLY29, GLY32, THR97, GLY99, PHE100, THR101 and ASN140. The results showed that o-hydroxyl structures are the active functional groups for potential inhibitors of *Pf*LDH.

Regarding artesunate, an artemisinin derivative is a sesquiterpene lactone containing an endoperoxide bridge that is essential for the mode of action. The cleavage of the peroxide bridge in the presence of ferrous ions (Fe^2+)^ from heme forms a rapid rearrangement to produce carbon-centered free radicals, leading to chemical modification and inhibition of a variety of parasite molecules, resulting in parasite death [42]. Interestingly, the docking results from this study revealed that artesunate also inhibited the *Pf*LDH enzyme by extensively interacting with the active site. Its endoperoxide bridge strongly interacted with ILE54 with two hydrogen bonds, ILE31, GLY29, GLY32, THR97 and GLY99, with a high binding energy (− 11.21 kcal/mol). CQ possessed a weak interaction by forming only one hydrogen bond with GLY99 with a binding energy of − 6.65 kcal/mol, which is similar to a previous report that it forms hydrogen bonds with ASP53 and GLY99 [9].

According to the docking results, GLY29, ILE31, GLY32, ASP53, GLY99, THR101 and ASN140 are the essential residues in the *Pf*LDH active site that participate in the interactions of active inhibitors. These results are in accordance with a previous study reporting that commercially available drugs, including itraconazole, atorvastatin and posaconazole (analogs of NADH, an essential cofactor of pLDH), showed the best docking energy values and fit well in the binding pocket of the *Pf*LDH active site. They interact with GLY29, MET30, ILE31, GLY32, ASP53, TYR85, THR97, GLY99, GLU122, ASN140 and SER245 [38].

The results supported the antimalarial activity, and active compounds that inhibited the growth of the *P. falciparum* K1 strain also strongly interacted with the residues in the NADH binding pocket located in the *Pf*LDH active site. Therefore, the mechanism of growth inhibition of *P. falciparum* by the active compounds results from competitive inhibition with NADH of the *Pf*LDH enzyme [38].

## Conclusions

The present study demonstrated that quercetin is a potential compound responsible for the antimalarial activity of *D. bulbifera* and is an inhibitor of *Pf*LDH. It appears to be an attractive compound for the development of new antimalarial agents. These findings provide more evidence to support the traditional use of *D. bulbifera* for malaria treatment. Structural models of its interactions at the *Pf*LDH active site are plausibly useful for the future design of antimalarial drugs.

## Data Availability

The data associated with this study are included in this published article. Additional files are available from the corresponding author upon reasonable request.

## Abbreviations

ACTs: Artemisinin-based combination therapies
ATP: Adenosine triphosphate
C: Carbon
CC_50_: 50% Cytotoxic concentration
COSY: Correlation Spectroscopy
CQ: Chloroquine
DEPT: Distortionless Enhancement by Polarization Transfer
DMSO: Dimethyl sulfoxide
EI-MS: Electron Ionization Mass Spectrometer
H: Proton
HMBC: Heteronuclear Multiple Bond Correlation
HMQC: Heteronuclear Multiple Quantum Coherence
IC_50_: The half maximal inhibitory concentration
LDH: Lactate dehydrogenase
N: Nitrogen
NADH: Nicotinamide adenine dinucleotide
MTT: 3-[4,5-dimethylthiazol-2-yl]-2,5-diphenyl-tetrazolium bromide
O: Oxygen
*Pf*LDH: *Plasmodium falciparum* lactate dehydrogenase
WHO: World Health Organization
